# PIKfyve preserves endolysosomal function in photoreceptors and RPE cells to maintain retinal integrity

**DOI:** 10.1038/s41419-026-08855-2

**Published:** 2026-05-16

**Authors:** Ammaji Rajala, Larissa J. Trevino, Thamaraiselvi Saravanan, Tyler M. Black, Mohd A. Bhat, Tuan Ngo, Mark Eminhizer, Jianhai Du, Visvanathan Ramamurthy, Raju V. S. Rajala

**Affiliations:** 1 Department of Ophthalmology, University of Oklahoma Health Sciences Center, Oklahoma City, OK USA; 2 OU Health Dean McGee Eye Institute, Oklahoma City, OK USA; 3 Department of Biochemistry and Molecular Medicine, West Virginia University Health Sciences, Morgantown, WV USA; 4 Department of Ophthalmology and Visual Sciences, West Virginia University Health Sciences, Morgantown, WV USA; 5 Department of Biochemistry and Physiology, University of Oklahoma Health Sciences Center, Oklahoma City, OK USA; 6 Department of Cell Biology, University of Oklahoma Health Sciences Center, Oklahoma City, OK USA

**Keywords:** Cellular neuroscience, Retina

## Abstract

Photoreceptors rely on efficient clearance of outer segment material and mislocalized proteins to maintain cellular health and visual function. While retinal pigment epithelial (RPE) cells remove shed outer segment tips through daily phagocytosis, the mechanisms by which photoreceptors eliminate misfolded or mistargeted proteins are not well understood. Here, we identify PIKfyve, a lipid kinase that synthesizes phosphatidylinositol 3,5-bisphosphate [PI(3,5)P₂], as a key regulator of degradative and metabolic pathways in the retina. PIKfyve is highly expressed in rod photoreceptors, and its selective deletion causes progressive retinal degeneration marked by vacuolation, increased lysosomal markers, loss of outer nuclear layer thickness, and decline of rod and cone function. Partial or complete reduction of PIKfyve further accelerates degeneration in P23H rhodopsin mutant mice. In the RPE, PIKfyve deficiency disrupts phagocytosis and autophagy, leading to the accumulation of rhodopsin, lysosomal proteins, and lipid droplets, accompanied by metabolic imbalance. These results demonstrate that PIKfyve is essential for maintaining photoreceptor and RPE integrity by supporting lysosomal function, protein turnover, and metabolic stability, and suggest that enhancing PIKfyve activity may offer therapeutic potential for retinal degenerative diseases. This study is timely, as pharmacological inhibition of PIKfyve with apilimod—currently under clinical investigation for autoimmune, neurodegenerative, and infectious diseases—raises significant safety concerns, including lysosomal swelling, vacuolization, and impaired protein degradation, which could ultimately compromise retinal homeostasis and vision.

**Figure:**
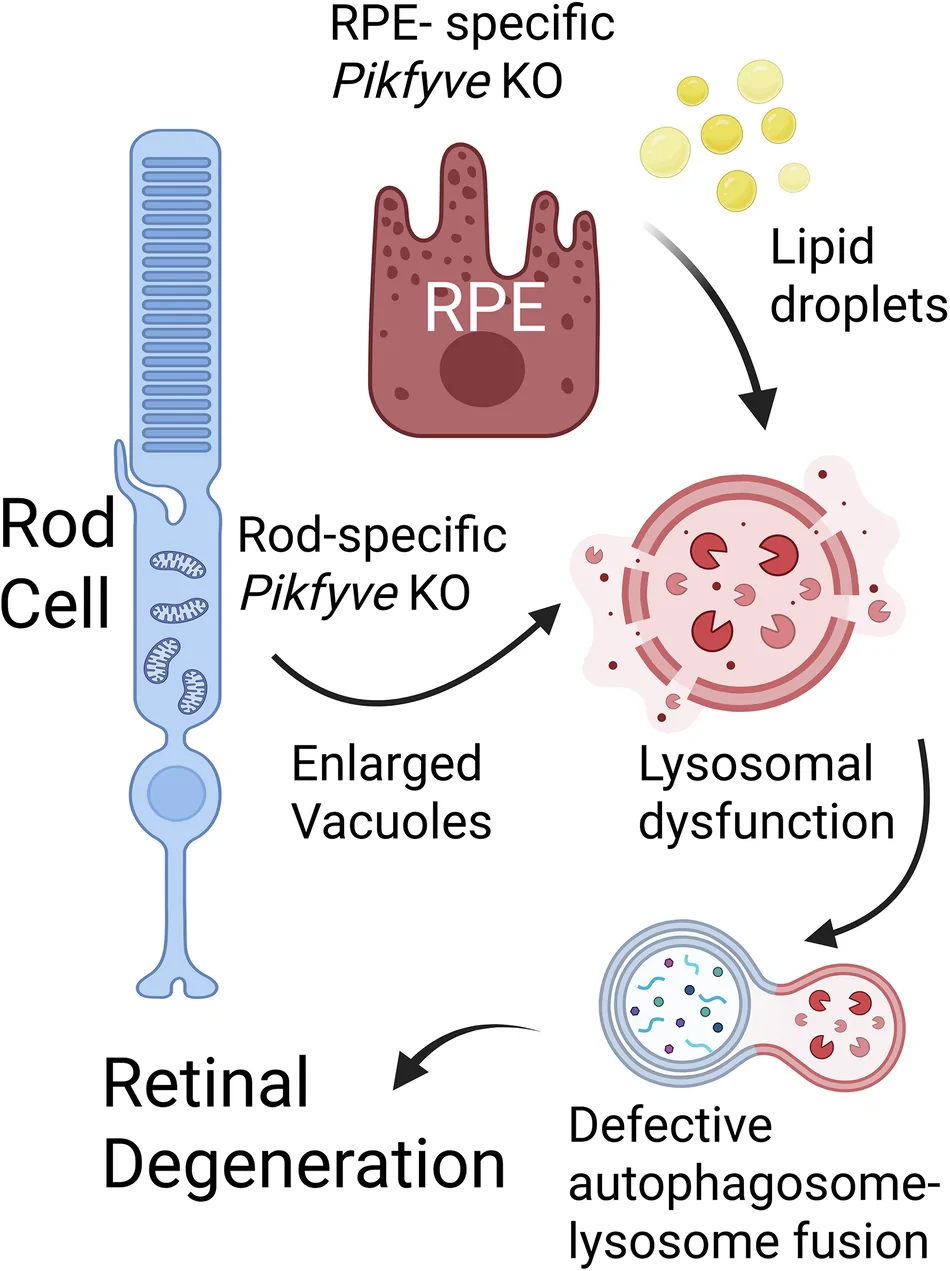

## Introduction

Phosphoinositides (PIPs) make up only a small fraction of cellular phospholipids, but are essential for nearly all aspects of cell function and survival [1–4]. Seven PIPs are formed through reversible phosphorylation, regulating key processes such as membrane trafficking, cytoskeletal organization, vesicle transport, ciliogenesis, and signal transduction [1–5]. Mutations in phosphoinositide (PI)-metabolizing enzymes cause severe neurological and ocular defects [6–8]. Several human diseases have been linked to genes encoding PI-converting enzymes and their effectors [6–13]. However, the specific role of disrupted PIP metabolism in these disorders remains poorly understood.

In the mammalian genome, approximately 50 genes encode PI kinases and phosphatases, which generate seven distinct PIPs [14]. Their distribution in retinal cell types was previously unknown. Using translating ribosome affinity purification (TRAP), we mapped the expression of PI-converting enzymes across retinal cells [15]. Our data indicate that one of the PI kinases, PIKfyve, is significantly enriched in rod, cone, and RPE cells compared to RGC and Müller glia. PIKfyve, a PI 5-kinase, converts phosphatidylinositol 3-phosphate [PI(3)P] into phosphatidylinositol 3,5-bisphosphate [PI(3,5)P₂] and also generates phosphatidylinositol 5-phosphate [PI(5)P] from phosphatidylinositol [6]. PIKfyve plays a key role in the biogenesis of early endosomes into late endosomes and lysosomes, processes vital for the breakdown of cellular waste and the recycling of materials [16, 17]. Mutations or loss of PIKfyve activity and its associated proteins are linked to neurological disorders marked by lysosomal enlargement and dysfunction [6]. In humans, heterozygous mutations in *PIKFYVE* have been associated with fleck corneal dystrophy [8].

The degradation of photoreceptor outer segment (OS) components is primarily mediated by retinal pigment epithelial (RPE) cells, which daily engulf approximately 10% of the distal OS tips [18]. Although traditionally described as phagocytosis, this process involves selective removal of OS fragments rather than ingestion of the entire OS and has features consistent with trogocytosis. In *Drosophila*, mislocalized rhodopsin is targeted for degradation via the endolysosomal system [19, 20]. *Drosophila* lack RPE, and defects in the endolysosomal pathway impair lysosomal function and contribute to retinal degeneration [19, 20]. In contrast, much less is understood about the cell-autonomous mechanisms photoreceptors use to clear mislocalized molecules, whether due to protein misfolding or defects in protein trafficking. Mislocalized or excess rhodopsin that fails to reach the OS is retained in the inner segment or cell body, where it is presumably targeted for degradation via the endolysosomal system. It has been previously shown that visual arrestin (ARR1) facilitates the internalization of rhodopsin through the clathrin-adaptor protein, AP-2, into the endolysosomal pathway [21]. In K296E mutant rhodopsin, abnormal ARR1–AP-2 complexes misroute rhodopsin and trigger photoreceptor cell death [21]. Our study demonstrates that PIKfyve regulates the endolysosomal pathway in photoreceptors and RPE cells, and its loss in rod photoreceptors leads to progressive retinal degeneration. Reduced PIKfyve expression further accelerated degeneration in P23H rhodopsin mutant mice.

## Results

### Enrichment of the FYVE finger-containing phosphoinositide kinase (PIKfyve) in rod, cone, and RPE cells

**Fig. 1:**
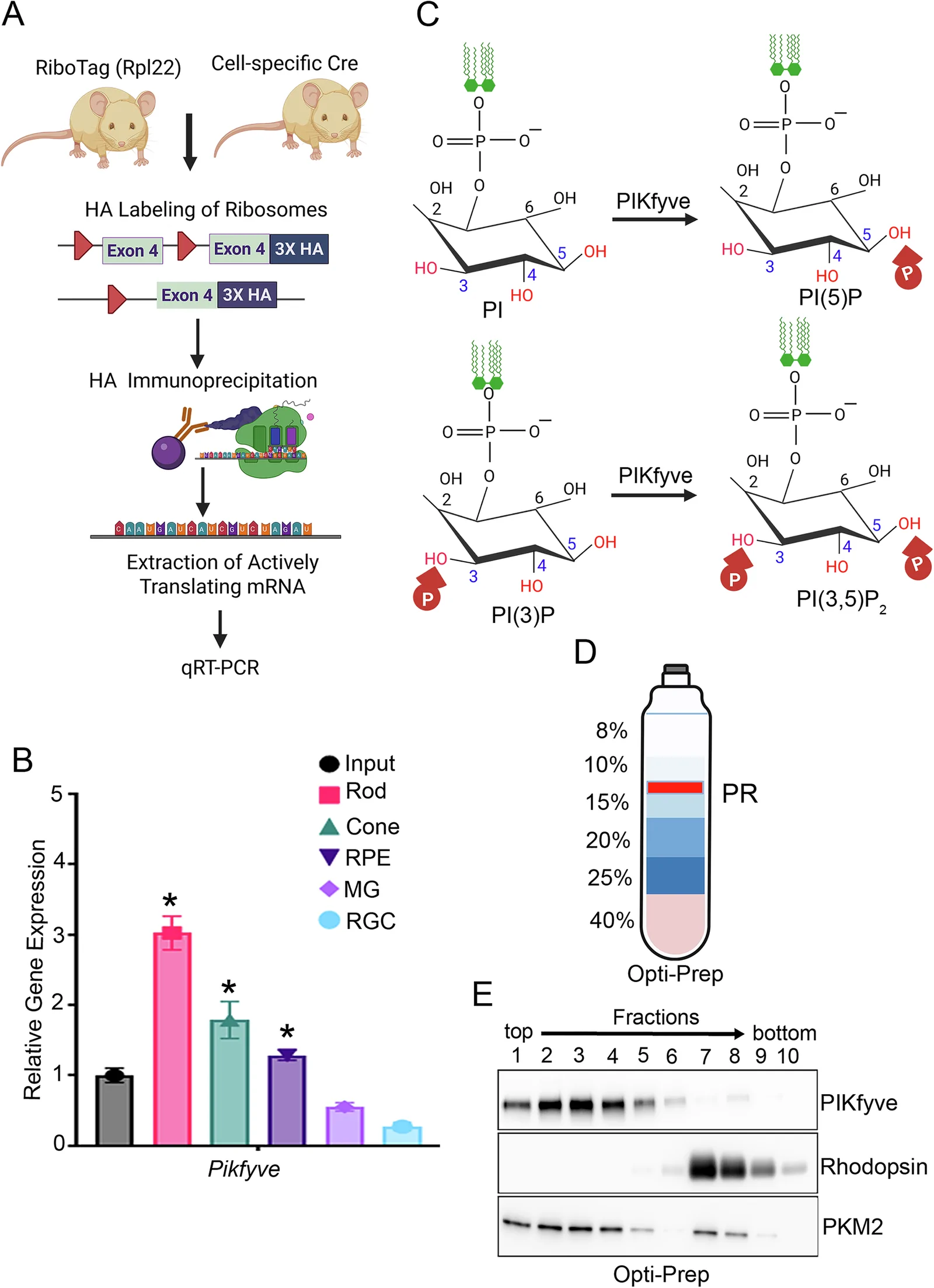
Expression of PIKfyve in retinal cells. A schematic of Translating Ribosome Affinity Purification (TRAP) is shown (**A**). Rpl22 mice were crossed with Cre-driver lines for rods, cones, RPE, Müller glia, and RGCs, followed by ribosome immunoprecipitation and mRNA isolation for PIKfyve expression analysis by qRT-PCR (**B**). qRT-PCR of Pikfyve mRNA from purified retinal cell types was normalized to *Rpl37* and *Rpl38* (Data *mean* *±* *SEM, n* = 3) and expressed relative to Input. PIKfyve is a phosphoinositide 5-kinase that converts PI to PI(5)P and PI(3)P to PI(3,5)P₂ (**C**). Opti-Prep (8–40%) gradient centrifugation (**D**) of retinal extracts separated intact photoreceptors (PR) and contents of broken inner segments, and the fractions were analyzed by immunoblotting for PIKfyve, rhodopsin, and PKM2 (**E**).

Using TRAP, we investigated the expression patterns of PI-converting enzymes specifically in retinal cells (Fig. 1A). We found that FYVE finger-containing PI 5-kinase (PIKfyve) was significantly enriched in rod, cone and RPE cells compared to total retina, Müller glia, and RGCs (Fig. 1B). PIKfyve phosphorylates PI to PI(5)P and PI(3)P to PI(3,5)P₂ (Fig. 1C). To confirm the rod-specific expression of PIKfyve, we performed Opti-Prep density gradient centrifugation on an 8–40% gradient (Fig. 1D) to isolate rod photoreceptors and broken rod inner segments, which release soluble cytoplasmic proteins [22]. The fractionation showed no significant co-migration between rhodopsin and PIKfyve, indicating that PIKfyve is localized in the inner segment, where most PI(3,5)P_2_ synthesis probably occurs (Fig. 1E). We previously reported that PKM2 is predominantly expressed in rod inner segments [22], where it also co-migrates with PIKfyve (Fig. 1E). Our study demonstrates that PIKfyve is present in rod photoreceptor cells.

### Rod-photoreceptor-specific deletion of PIKfyve and its impact on rod structure and function

**Fig. 2:**
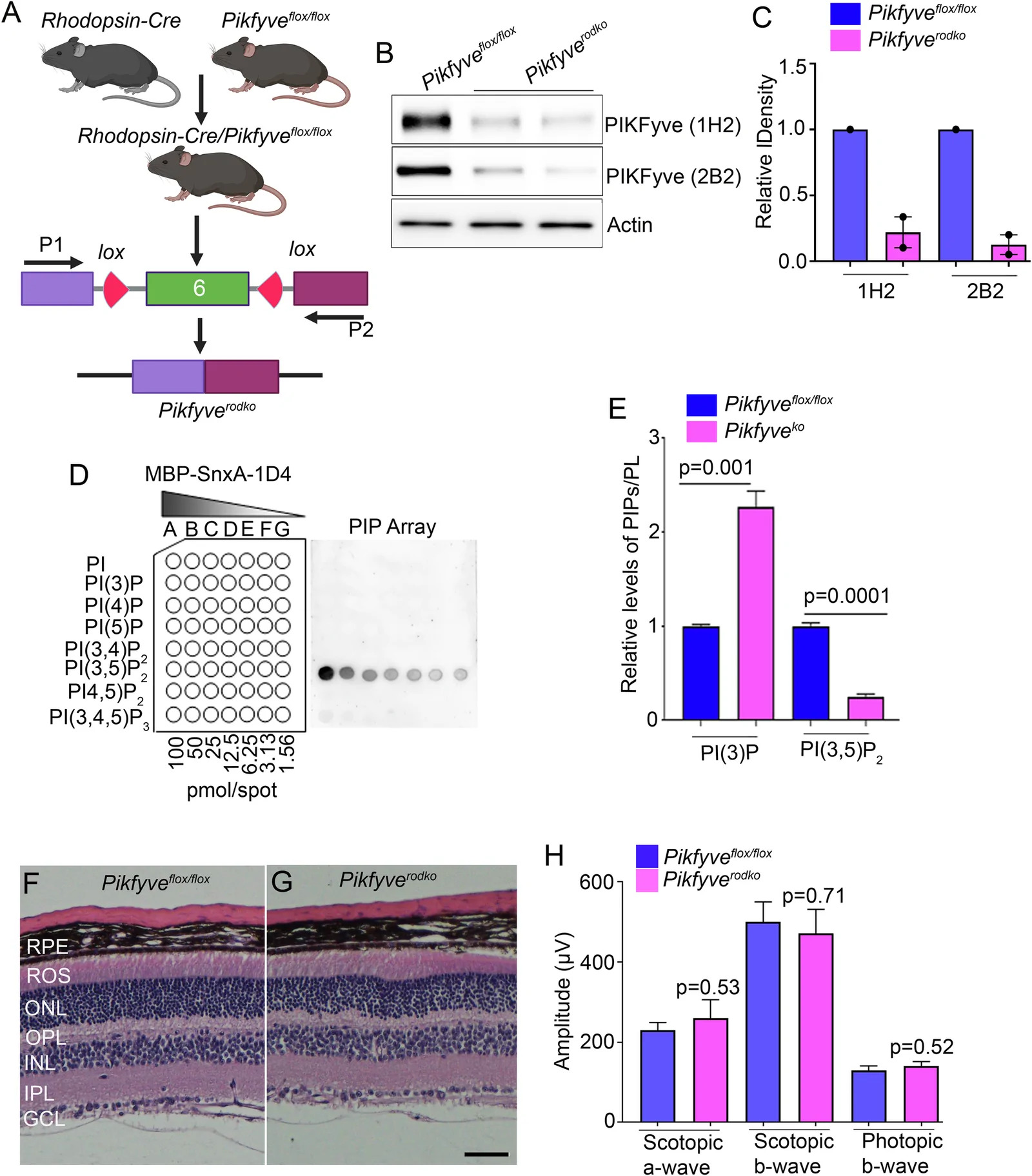
Generation and characterization of rod-specific PIKfyve knockout mice. Mice carrying floxed PIKfyve alleles (exon 6) were crossed with rhodopsin-Cre mice to generate rod-specific knockout (**A**). Genotypes were confirmed by PCR using primers P1 and P2. Retinal lysates from *Pikfyve*^*flox/flox*^ and *Pikfyve*^*rodko*^ mice were immunoblotted with two PIKfyve antibody clones (IH2, 2B2) and actin (**B**), and the data were normalized to actin (**C**). Data are mean ± *SEM (n* = 2*)*. The PIP-Array demonstrated the specificity of SnxA probe towards PI(3,5)P₂ (**D**). Phosphoinositide lipids were extracted from control and KO mouse retina, and the levels of PI(3)P and PI(3,5)P_2_ were measured (**E**). Data are mean ± *SEM, (n* = 3*)*. H&E-stained retinal sections from 4-week-old *Pikfyve*^*flox/flox*^ and *Pikfyve*^*rodko*^ mice (**F**, **G**). Scale bar = 50 µm. ERG analysis (scotopic a-wave, scotopic b-wave, and photopic b-wave amplitudes) was performed on 4-week-old *Pikfyve*^*flox/flox*^ and *Pikfyve*^*rodko*^ mice (**H**). Data are mean ± *SEM (n* = 12*)*.

Global deletion of PIKfyve is embryonically lethal [23]. Thus, we generated rod-specific PIKfyve knockout (*Pikfyve*^*rodko*^) mice using the rhodopsin promoter to drive Cre recombinase expression in rod photoreceptors (Fig. 2A). Four-week-old *Pikfyve*^*rodko*^ mouse retinas show more than 80% reduction in PIKfyve protein compared to control (*Pikfyve*^*flox/flox*^) retina (Fig. 2B, C). Recently, SnxA protein from the ameba *Dictyostelium discoideum* has been identified as a highly selective PI(3,5)P_2_-binding protein and characterized as a reporter for PI(3,5)P_2_ in both *Dictyostelium* and mammalian cells [24]. We cloned and expressed SnxA as a fusion protein with an N-terminal maltose-binding protein (MBP) tag and a C-terminal rhodopsin 1D4 tag. The MBP tag allows purification of the fusion protein from bacteria by binding to amylose beads, and the 1D4 tag is used to detect the interaction of the fusion protein with PI(3,5)P₂. A PIP array confirmed SnxA’s specific binding to PI(3,5)P_2_ in a concentration-dependent manner (Fig. 2D). We used SnxA to measure PI(3,5)P_2_ and 2XHRS probe to assess PI(3)P levels [25] on ELISA in *Pikfyve*^*flox/flox*^ (littermate control) and *Pikfyve*^*ko*^ retinas. We found significantly increased PI(3)P and decreased PI(3,5)P_2_ levels in *Pikfyve*^*ko*^ mouse retinas compared to *Pikfyve*^*flox/flox*^ mice (Fig. 2E). The PI(3,5)P_2_ levels decreased by 80%. In contrast, PI(3)P levels increased 2.5-fold compared to *Pikfyve*^*flox/flox*^ mice (Fig. 2E). Histological examination of 4-week-old *Pikfyve*^*rodko*^ mice showed no morphological changes compared to *Pikfyve*^*flox/flox*^ mice (Fig. 2F, G). We assessed retinal function in 4-week-old *Pikfyve*^*flox/flox*^ and *Pikfyve*^*rodko*^ mice using electroretinography (ERG). We found no significant differences in rod (scotopic a-wave and scotopic b-wave amplitudes) or cone (photopic b-wave amplitudes) function in *Pikfyve*^*rodko*^ mice compared to *Pikfyve*^*flox/flox*^ mice (Fig. 2H).

**Fig. 3:**
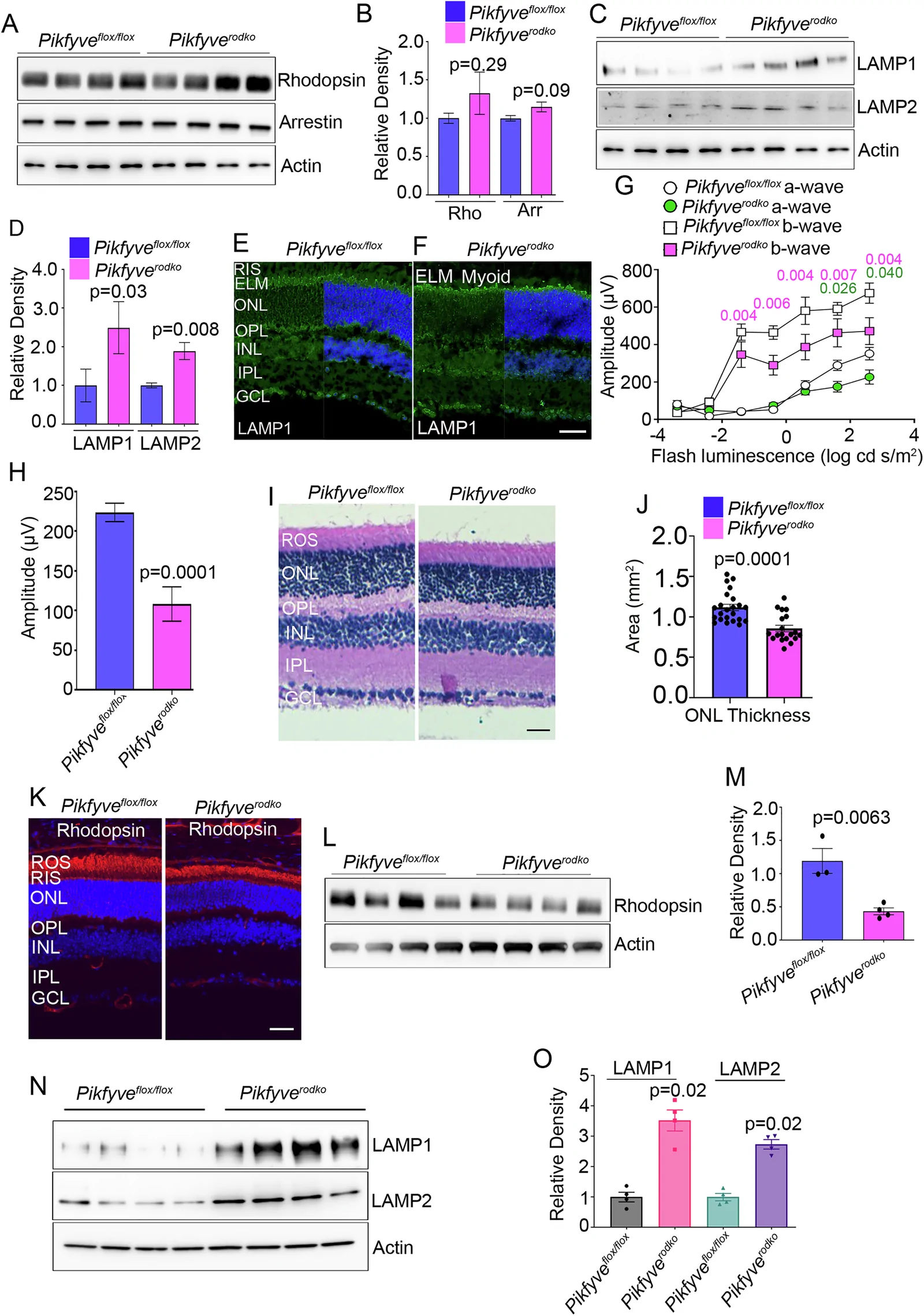
Age-dependent changes in rods lacking PIKfyve. Four-week-old *Pikfyve*^*flox/flox*^ and *Pikfyve*^*rodko*^ mouse retinal proteins were subjected to immunoblot analysis with rod photoreceptor markers (rhodopsin, arrestin) (**A**), lysosomal markers (LAMP1, LAMP2) (**C**), and normalized to actin (**B**, **D**). Data are mean ± *SEM (n* = 4*)*. Retinal sections from 4-week-old *Pikfyve*^*flox/flox*^ and *Pikfyve*^*rodko*^ mice were stained with LAMP1 antibody (**E**, **F**). ERG was performed on 14-week-old *Pikfyve*^*flox/flox*^ and *Pikfyve*^*rodko*^ mice to examine rod (scotopic a- and scotopic b-wave amplitudes) (**G**) and cone (photopic b-wave amplitudes) (**H**) function. Data are mean ± *SEM (n* = 8*)*. ERG responses were analyzed using a linear mixed-effects model, followed by the two-stage linear step-up procedure of Benjamini, Krieger, and Yekutieli for multiple-comparison testing. The *q* values are shown in the graphs. H&E-stained sections of 14-week-old *Pikfyve*^*flox/flox*^ and *Pikfyve*^*rodk*o^ mice (**I**). Scale bar = 50 µm. Quantification of ONL thickness using NIH ImageJ software confirms a significant decrease in *Pikfyve*^*rodk*o^ mice compared with *Pikfyve*^*flox/flox*^ mice (**J**). Data are mean ± *SEM (n* = 20*)*. Rhodopsin expression (**K**) and rhodopsin protein levels (**L**, **M**) were also lower in 14-week-old *Pikfyve*^*rodk*o^ mice compared with *Pikfyve*^*flox/flox*^ mice. Retina lysates from 16-week-old *Pikfyve*^*flox/flox*^ and *Pikfyve*^*rodk*o^ mice were immunoblotted with antibodies against LAMP1, LAMP2, and actin (**N**). Densitometry values were normalized to actin (**O**). Data are mean ± *SEM (n* = 4*)*. The actin blot shown in (**A**, **C**) is the same.

At 4 weeks, rod photoreceptor marker proteins, rhodopsin and rod arrestin levels remain unchanged in *Pikfyve*^*rodko*^ retinas, which are comparable to *Pikfyve*^*flox/flox*^ mice (Fig. 3A, B). However, we found significantly increased levels of lysosomal markers, LAMP1 and LAMP2, in *Pikfyve*^*rodko*^ mouse retina compared to *Pikfyve*^*flox/flox*^ mouse retina (Fig. 3C, D). To rule out whether the increased LAMP1 observed in immunoblots originates from Müller glia or microglia, retina sections from 4-week-old *Pikfyve*^*rodko*^ and *Pikfyve*^*flox/flox*^ mice were stained with a LAMP1 antibody. The results show increased LAMP1 expression in the external limiting membrane (ELM), located between the photoreceptor inner segments and Müller glial cells, in *Pikfyve*^*rodko*^ mice compared to *Pikfyve*^*flox/flox*^ mice (Fig. 3E, F).

By 16 weeks, *Pikfyve*^*rodko*^ mice exhibited significantly reduced rod (scotopic a-wave and scotopic b-wave amplitudes) (Fig. 3G) and cone (photopic b-wave amplitude) function compared to *Pikfyve*^*flox/flox*^ mice (Fig. 3H). Additionally, *Pikfyve*^*rodko*^ mice showed marked thinning of the outer nuclear layer (ONL) (Fig. 3I, J), reduced rhodopsin expression (Fig. 3K), and lower rhodopsin protein levels (Fig. 3L, M) compared to *Pikfyve*^*flox/flox*^ mice. The LAMP1 and LAMP2 levels were significantly higher in 16-week-old *Pikfyve*^*rodko*^ compared to *Pikfyve*^*flox/flox*^ mouse retina (Fig. 3N, O), indicating lysosomal dysfunction.

**Fig. 4:**
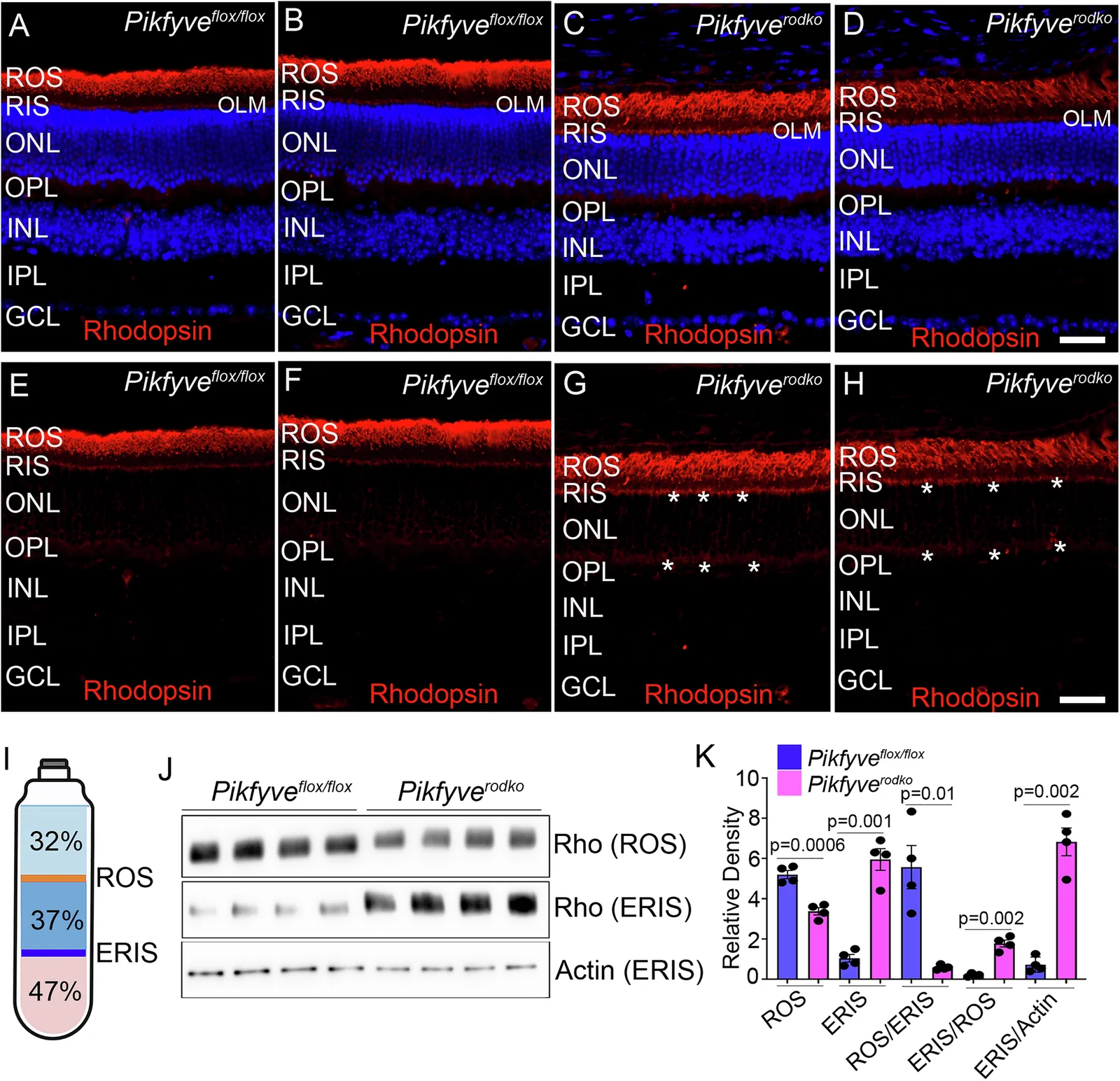
Mislocalization of rhodopsin in *Pikfyve*^*rodko*^ mice. Retinal sections from 14-week-old *Pikfyve*^*flox/flox*^ (**A**, **B**, **E**, **F**) and *Pikfyve*^*rodk*o^(**C**, **D**, **G**, **H**) mice were immunostained with rhodopsin and DAPI (**A**–**D**). **E**–**H** show rhodopsin staining alone. Scale bar = 50 µm. *Asterisks indicate mislocalized rhodospin in the RIS and OPL. ROS rod outer segments, RIS rod inner segments, OLM outer limiting membrane, ONL outer nuclear layer, OPL outer plexiform layer, INL inner nuclear layer, IPL inner plexiform layer, GCL ganglion cell layer. Rod outer segments (ROS) and enriched rod inner segments (ERIS) were isolated from 2-month-old *Pikfyve*^*flox/flox*^ and *Pikfyve*^*rodk*o^ mice using discontinuous sucrose density gradient centrifugation (**I**). Four independent ROS (50 ng) and ERIS (100 ng) fractions were immunoblotted for rhodopsin and actin (**J**). The actin immunoblot was performed using 3 µg of ERIS protein. Quantification is presented as ROS, ERIS, ROS/ERIS, ERIS/ROS, and ERIS/actin (**K**). Data are mean ± *SEM (n* = 4*)*.

We observed mislocalization of rhodopsin in *Pikfyve*^*rodko*^ mice (Fig. 4C, D, G, H). Rhodopsin expression was reduced, with rhodopsin accumulating at the outer limiting membrane and additional rhodopsin signal appearing in the outer plexiform layer in *Pikfyve*^*rodko*^ mouse retina, compared to *Pikfyve*^*flox/flox*^ mouse retina (Fig. 4A, B, E, F). To assess and quantify rhodopsin mislocalization, we separated rod outer segments (ROS) and enriched rod inner segments (ERIS) from 2-month old *Pikfyve*^*flox/flox*^ and *Pikfyve*^*rodko*^ mouse retinas using discontinuous sucrose gradient centrifugation [26] (Fig. 4I). This method yields pure ROS and ERIS containing inner segments with other retinal cells; these fractions were previously validated with RPE, outer segment, and inner segment markers [27]. Immunoblot analysis showed significantly reduced rhodopsin in the ROS fraction and increased rhodopsin in the ERIS fraction of *Pikfyve*^*rodko*^ mice compared to *Pikfyve*^*flox/flox*^ mice (Fig. 4J, K). After normalizing rhodopsin in the ERIS fraction to ROS and actin, rhodopsin levels were increased in ERIS from *Pikfyve*^*rodko*^ retinas compared with *Pikfyve*^*flox/flox*^ retinas, indicating mislocalization of rhodopsin to the inner segments.

### Deletion of PIKfyve in rod photoreceptor cells results in vacuolation and lysosomal enlargement

**Fig. 5:**
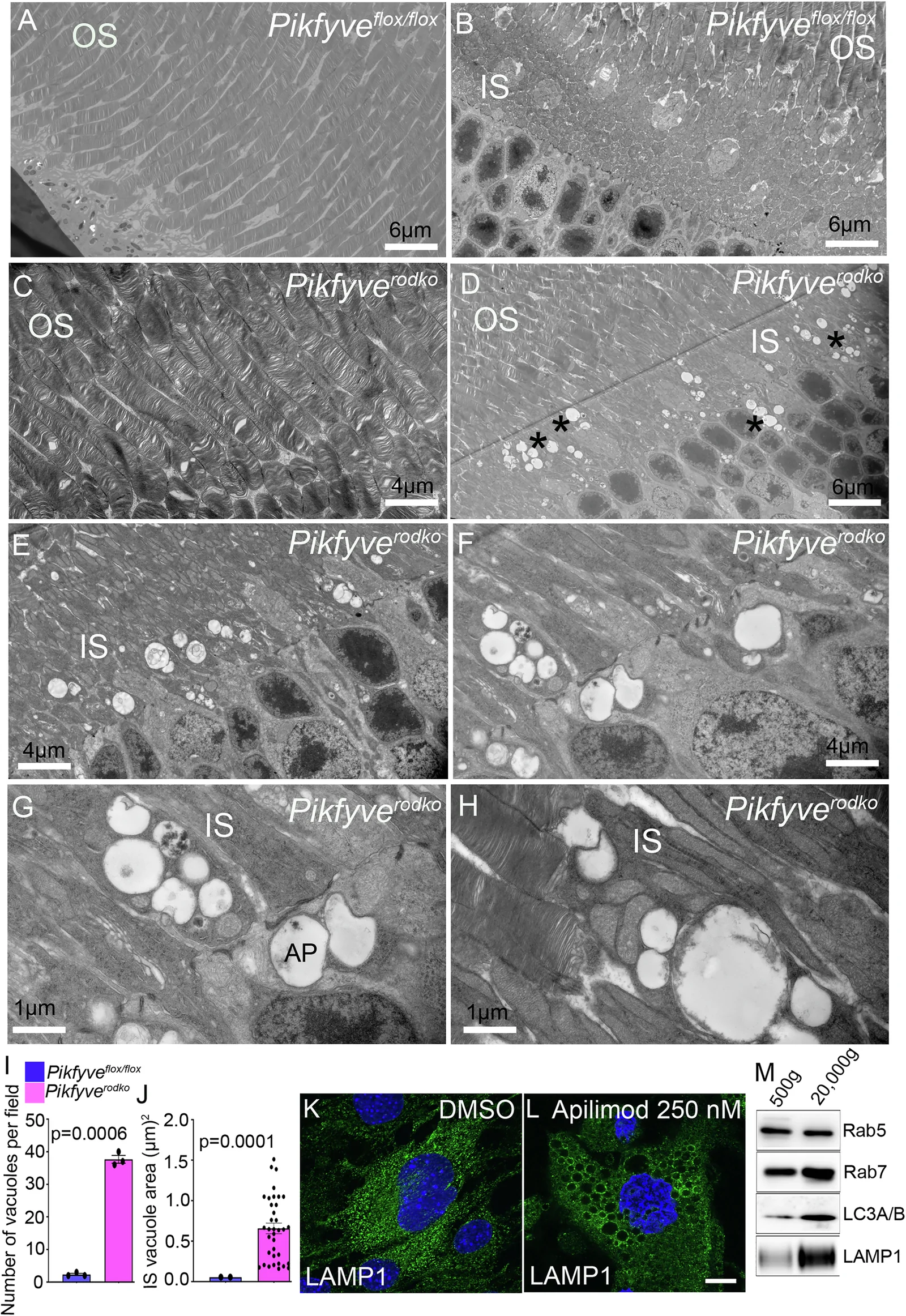
Abnormal vacuolization in *Pikfyve*^*rodko*^ mice. Mouse retinal tissues from a 14-week-old *Pikfyve*^*flox/flox*^ (**A**, **B**) and *Pikfyve*^*rodko*^ (**C**–**E**) were analyzed by TEM. **F**–**H** display a higher magnification of the region shown in (**E**). Inner segment vacuoles were quantified per field (WT: 2–3; KO: 36–40) (**I**). Data are mean ± *SEM, (n* = 3*)*. The size of the vacuoles was measured as cross-sectional area (WT mean 0.0555 µm²; KO mean 0.673 µm²) (**J**). Data are mean ± *SEM, (n* = 30*)*. DMSO- and 250 nM Apilimod-treated 661 W cells were stained with LAMP1 antibody (**K**, **L**). Isolated vacuoles (20,000g fraction) and input (500g fraction) were immunoblotted using Rab5, Rab7, LC3A/B, and LAMP1 antibodies (**M**). OS outer segment, IS inner segment, *vacuoles, AP autophagosome.

To examine potential ultrastructural defects in *Pikfyve*^*rodko*^ mice, we performed transmission electron microscopy (TEM) on retinal tissue from 14-week-old *Pikfyve*^*rodko*^ and *Pikfyve*^*flox/flox*^ mice. In *Pikfyve*^*flox/flox*^ mouse retina, photoreceptor outer segments showed tightly packed, organized membranous discs, while the inner segments contained elongated mitochondria and preserved cytoplasmic architecture (Fig. 5A, B, Fig. S1). In *Pikfyve*^*flox/flox*^ retinas, outer segment length and alignment were largely preserved, with mild focal irregularities in disc stacking (Fig. 5C, Fig. S1). In contrast, large membrane-bound, electron-lucent vacuoles accumulated within the inner segment cytoplasm of *Pikfyve*^*Rodko*^ photoreceptors (Fig. 5D–F, Fig. S1). These vacuoles varied in size and often contained heterogeneous or partially degraded material, suggesting they may be organelles such as late endosomes, autolysosomes, or incompletely fused autophagic vesicles, indicative of blocked autophagic flux and disrupted endolysosomal maturation (Fig. 5G, H, Fig. S1). TEM revealed a marked increase in inner segment vacuolization in KO retinas compared with WT controls. WT inner segments contained few vacuoles (2–3 per field) with a mean cross-sectional area of 0.0555 µm². In contrast, KO inner segments exhibited substantial vacuole accumulation (36–40 per field) (Fig. 5I), and vacuole size was markedly increased, with a mean area of 0.673 µm² (range 0.159–1.511 µm²) (Fig. 5J). These data demonstrate a pronounced increase in both vacuole number and size in KO photoreceptors relative to WT controls. These ultrastructural changes suggest that PIKfyve plays a crucial role in maintaining endolysosomal integrity and autophagic processing in photoreceptors, and its loss may lead to progressive degeneration associated with the buildup of incompletely degraded vesicular material.

To investigate the contents of enlarged vacuoles caused by PIKfyve deletion, we treated 661 W cells, derived from mouse cone photoreceptor cells [22], with the PIKfyve inhibitor Apilimod for 60 min at 37°C. After treatment, both DMSO control and Apilimod-treated cells were fixed and stained with a LAMP1 antibody. The results showed that the enlarged vacuoles in Apilimod-treated cells were positively stained for LAMP1 (Fig. 5K, L). LAMP1 is commonly recognized as a lysosomal marker, but it can also be found on late endosomes, phagosomes, and autophagosomes [28]. We isolated vacuoles from 661 W cells treated with Apilimod using a method that uses sorbitol to loosen cell membranes [29]. Immunoblot analysis of the input fraction (500 g) and isolated vacuoles (20,000 g) was performed using markers for early endosomes (Rab5), late endosomes (Rab7), autophagy (LC3A/B), and lysosomes (LAMP1). The results showed enrichment of Rab7 and LC3A/B in the vacuoles compared to the input, indicating the presence of amphisomes (late endosomes and autophagosomes) (Fig. 5M). Additionally, LAMP1 levels were significantly higher in the vacuoles than in the input (Fig. 5M). These findings suggest that the absence of PIKfyve impairs the fusion of late endosomes and autophagosomes with lysosomes.

**Fig. 6:**
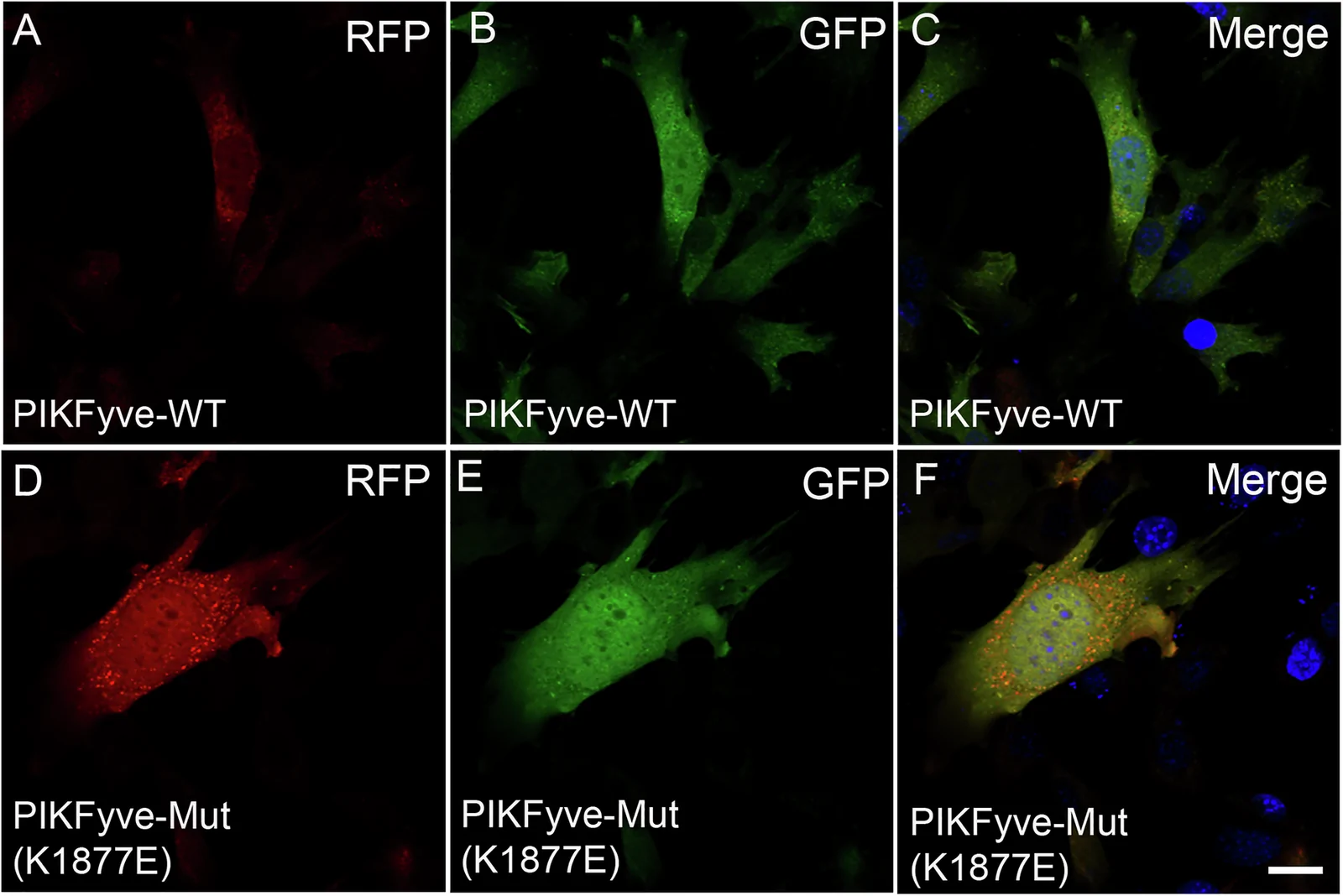
Autophagic flux analysis in 661 W photoreceptor cells. 661 W cells were transduced with adenovirus 5 (Ad5)–tandem GFP-RFP–LC3 construct (Vector Biolabs) and co-expressed either wild-type PIKfyve (**A**–**C**) or PIKfyve mutant K1877E (**D**–**F**). RFP (**A**, **D**), GFP (**B**, **E**), and merged images (**C**, **F**). Scale bar, 50 µm.

To determine whether the vesicular accumulation observed in *Pikfyve*^*rodko*^ photoreceptors reflects impaired autophagic maturation, we examined autophagic progression using the pH-sensitive Ad5–tandem GFP-RFP–LC3 reporter in 661 W cells expressing either wild-type PIKfyve (Fig. 6A–C) or the catalytically inactivated PIKfyve mutant K1877E [30] (Fig. 6D–F). In the tandem reporter, autophagosomes appear yellow due to combined GFP and RFP fluorescence at neutral cytoplasmic pH, whereas autolysosomes appear red only because the acidic lysosomal environment quenches GFP fluorescence while RFP remains stable [31]. Cells expressing wild-type PIKfyve showed predominantly diffuse LC3 fluorescence with few puncta and minimal vacuolation (Fig. 6A–C). In contrast, expression of PIKfyve mutant K1877E was associated with enlarged cell morphology and accumulation of yellow LC3-positive puncta (Fig. 6D–F), consistent with increased LC3-positive, non-acidified autophagic vesicles. These findings are consistent with the TEM data demonstrating accumulation of membrane-bound, electron-lucent vacuoles in *Pikfyve*^*rodko*^ inner segments and with the enrichment of LAMP1, Rab7, and LC3A/B in apilimod-induced vacuoles. Together, these results support disruption of endolysosomal maturation and autophagic processing following PIKfyve loss-of-function.

Interestingly, RPE from 4-month-old *Pikfyve*^*rodko*^ mice showed significantly reduced levels of retinal pigment epithelial protein 65 (RPE65) compared with RPE from *Pikfyve*^*flox/flox*^ mice (Fig. S2A, B). In contrast, the lysosomal markers LAMP1 and LAMP2, as well as the autophagy marker LC3A/B, showed no significant differences between *Pikfyve*^*flox/flox*^ and *Pikfyve*^*rodko*^ RPE (Fig. S2A, B). The TEM analysis of *Pikfyve*^*rodko*^ RPE shows an increased number of electron-dense vesicles consistent with accumulated phagosomes or phagolysosomes, along with slightly more dispersed melanosomes (Fig. S3B). Small dense bodies, likely representing membrane debris or early degradative intermediates, are also more apparent. In contrast, wild-type RPE displays a more orderly cytoplasm with fewer phagosomes and well-positioned melanosomes (Fig. S3A). These findings suggest a mild disruption in phagocytic processing and intracellular organization in the absence of PIKfyve in rods.

### Loss of PIKfyve in rods exacerbates retinal degeneration in P23H rhodopsin mutant mice

**Fig. 7:**
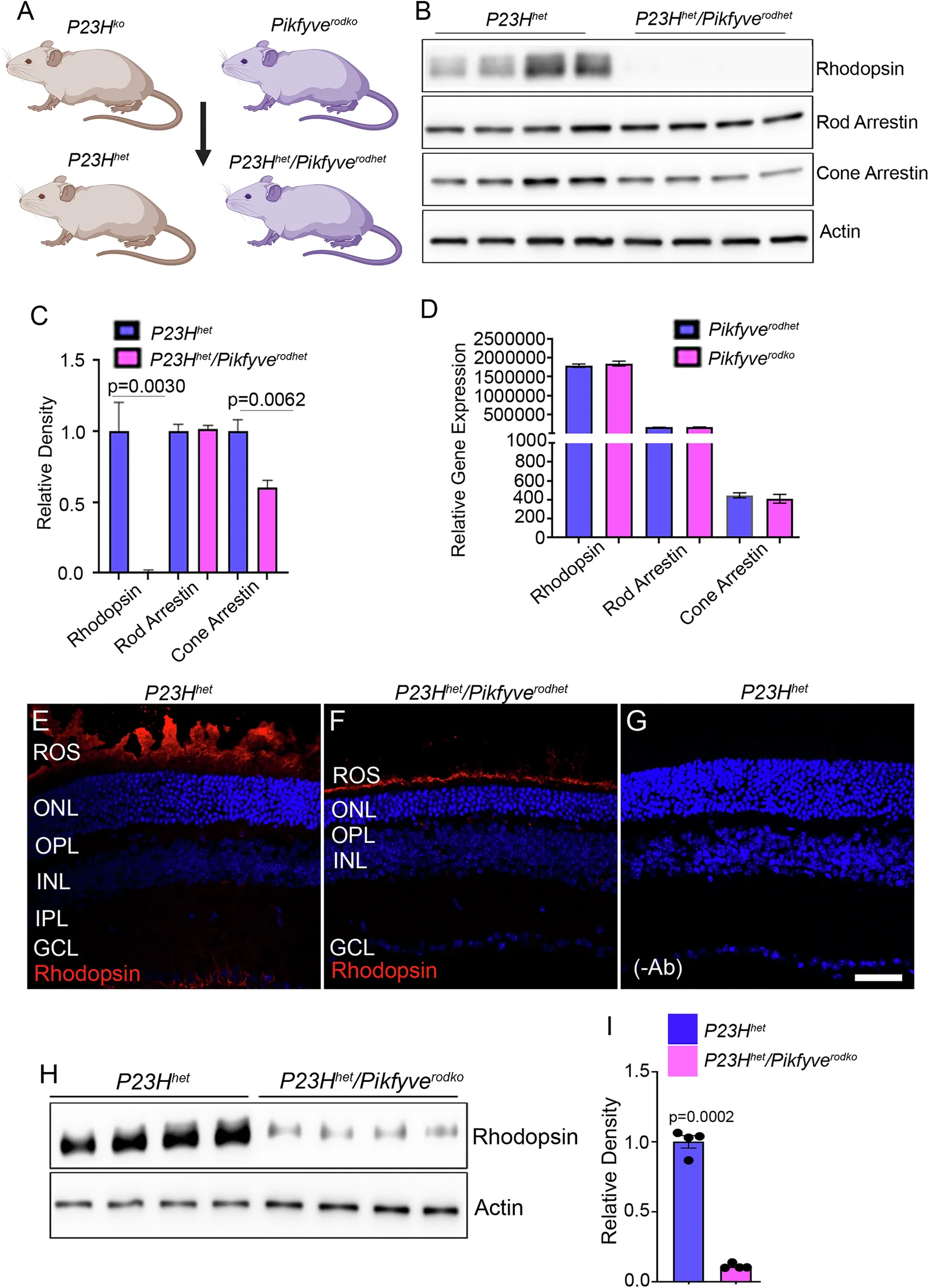
Loss of PIKfyve in rods exacerbates retinal degeneration in P23H rhodopsin mutant mice. We bred P23H homozygous mice with *Pikfyve*^*rodko*^ mice to generate *P23H*^*het*^ and *P23H*^*het*^/*Pikfyve*^*rodhet*^ mice (**A**). Retina lysates from 1-month-old *P23H*^*het*^ mice and *P23H*^*het*^*/Pikfyve*^*rodhet*^ mice were immunoblotted with rhodopsin, rod arrestin, cone arrestin, and actin antibodies (**B**), and their densities were normalized to actin (**C**). Data are mean ± *SEM*, (*n* = 4). TRAP on 2-month-old *Pikfyve*^*rodhet*^ and *Pikfyve*^*rodko*^ mouse retinas, followed by RNA sequencing, shows no change in the expression levels of rhodopsin, rod arrestin, and cone arrestin between the two genotypes (**D**). Data are mean ± *SEM*, (*n* = 3). Retinal cryo sections from *P23H*^*het*^ (**E**) and *P23H*^*het*^*/Pikfyve*^*rodhet*^ (**F**) mice were stained with rhodopsin antibody. **G** shows the omission of the primary antibody. Retina lysates from 1-month-old *P23H*^*het*^ mice and *P23H*^*het*^*/Pikfyve*^*rodko*^ mice were immunoblotted with rhodopsin and actin antibodies (**H**), and rhodopsin density was normalized to actin (**I**). Data are mean ± *SEM*, (*n* = 4).

P23H rhodopsin mutant mice are a widely used animal model for autosomal dominant retinitis pigmentosa (adRP) [32]. This model features a proline-to-histidine substitution at position 23 (P23H) in the rhodopsin (RHO) gene—a common mutation associated with autosomal dominant retinitis pigmentosa (adRP) in humans [32]. Morphological analysis of the retina revealed that at postnatal day 63 (P63), the ONL consisted of 9–10 rows of nuclei in WT, 6–7 rows in P23H heterozygous, and 0–1 row in homozygous P23H mice [32]. To assess whether reduced PIKfyve increases the susceptibility of already vulnerable photoreceptors to degeneration, and to investigate the role of PIKfyve in rod degeneration in P23H mice, we crossed P23H homozygous mice with *Pikfyve*^*rodko*^ mice to generate *P23H*^*het*^ and *P23H*^*het*^/*Pikfyve*^*rodhet*^ mice (Fig. 7A). When we examined rhodopsin levels, we observed an absence of rhodopsin, significantly lower levels of cone arrestin, and no change in rod arrestin levels in *P23H*^*het*^*/Pikfyve*^*rodhet*^ mice compared to *P23H*^*het*^ mice (Fig. 7B, C). TRAP on 2-month-old *Pikfyve*^*rodhet*^ and *Pikfyve*^*rodko*^ mouse retinas, followed by RNA sequencing, shows no change in the mRNA expression levels of rhodopsin, rod arrestin, and cone arrestin between the two genotypes (Fig. 7D). Consistent with decreased levels of rhodopsin on immunoblotting, immunohistochemistry further confirms reduced expression of rhodopsin in *P23H*^*het*^*/Pikfyve*^*rodhet*^ compared to *P23H*^*het*^ mice (Fig. 7E–G). The ONL contained 11 to 12 rows of photoreceptor nuclei, which is consistent with the normal range observed in rodents lacking retinal degeneration [33]. In the 1-month-old *P23H*^*het*^*/Pikfyve*^*rodhet*^ mouse retina, we observed approximately 5-6 rows of photoreceptor nuclei, indicating retinal degeneration (Fig. 7E, F). These observations suggest that the loss of one PIKfyve copy in P23H mouse rods exacerbates both rod and cone degeneration. Loss of both PIKfyve copies in the rods of *P23H*^*het*^ mice also led to a marked decrease in rhodopsin levels compared to *P23H*^*het*^ controls (Fig. 7H, I).

### Studies on PIKfyve deletion in the retinal pigment epithelium

**Fig. 8:**
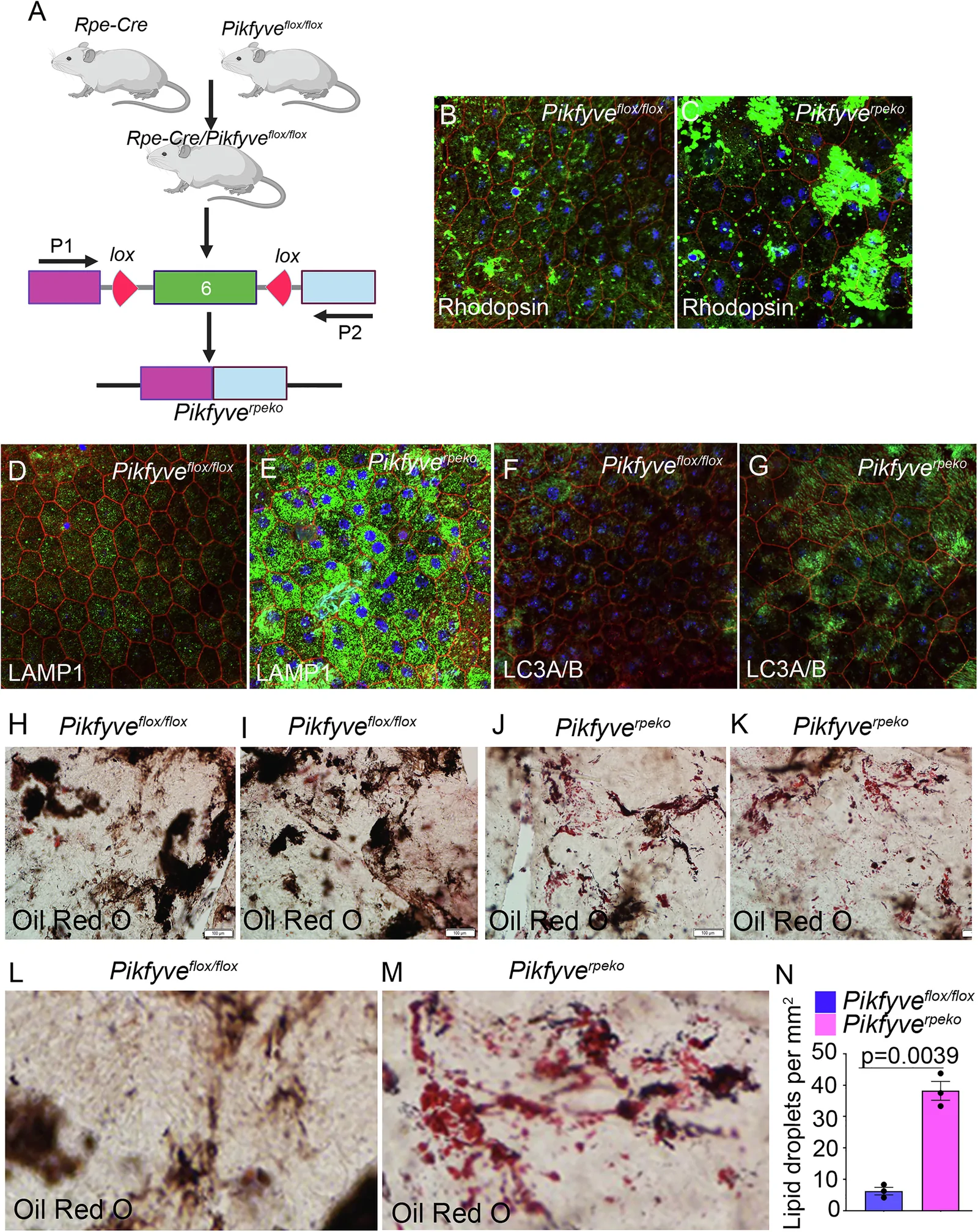
Generation and characterization of RPE-specific PIKfyve knockout mice. Mice with floxed PIKfyve alleles flanking exon 6 were bred with mice expressing Cre recombinase under the control of the bestrophin (BEST1) promoter (**A**) to produce RPE-specific PIKfyve KO mice (*Pikfyve*^*rpeko*^). P1 and P2 are primer locations to identify desired genotypes. Flat mounts were prepared from *Pikfyve*^*flox/flox*^ and *Pikfyve*^*rpeko*^ mice, then stained with antibodies for rhodopsin (**B**, **C**), LAMP1 (**D**, **E**), and LC3A/B (**F**, **G**). Oil Red O staining of RPE flatmounts from 2-month-old *Pikfyve*^*flox/flox*^ (**H**, **I**) and *Pikfyve*^*rpeko*^ (**J**, **K**) mice. **L**, **M** show higher magnifications of (**H**) (*Pikfyve*^*flox/flox*^) and (**K)** (*Pikfyve*^*rpeko*^). Oil Red O–positive lipid droplets were quantified using ImageJ software (**N**). Data are mean ± *SEM (n* = 3*)*.

Our TRAP data also reveal increased PIKfyve expression in the RPE. Thus, we deleted PIKfyve in the RPE using Cre under the control of the RPE-specific bestrophin 1 promoter (Fig. 8A) and examined the effect of PIKfyve loss on the RPE. Flat mounts were prepared from 2-month-old RPE-specific PIKfyve KO mice (*Pikfyve*^*rpeko*^) and littermate controls (*Pikfyve*^*flox/flox*^) and stained with rhodopsin, LAMP1, and LC3A/B antibodies. The results indicate increased rhodopsin (Fig. 8B, C), LAMP1 (Fig. 8D, E), and LC3A/B (Fig. 8F, G) staining in *Pikfyve*^*rpeko*^ mouse RPE compared to *Pikfyve*^*flox/flox*^ mice. The increased rhodopsin expression suggests a slowdown in RPE phagocytosis, and the increased LAMP1 and LC3A/B expression indicate a defect in lysosomal autophagy and LC3-mediated phagocytosis. Oil Red O is a fat-soluble dye used for staining neutral lipids and triglycerides in cells and tissues [34], and increased Oil Red O-positive staining correlates with disease severity, particularly in models that mimic dry age-related macular degeneration (AMD) [35]. We observed increased Oil Red O staining in the RPE of 2-month-old *Pikfyve*^*rpeko*^ mice (Fig. 8J, K, M-higher magnification) compared to *Pikfyve*^*flox/flox*^ mice (Fig. 8H, I, L-higher magnification). Quantification of Oil Red O–positive cells revealed a significant increase in lipid droplets in the RPE of *Pikfyve*^*rpeko*^ mice compared to *Pikfyve*^*flox/flox*^ controls (Fig. 8N). Interestingly, 6-month-old *Pikfyve*^*rpeko*^ mice show a trend toward reduced rod function but not cone function compared with *Pikfyve*^*flox/flox*^ mice; however, these differences are not statistically significant (Fig. S4).

### Effect of PIKfyve loss on retinal metabolism

**Fig. 9:**
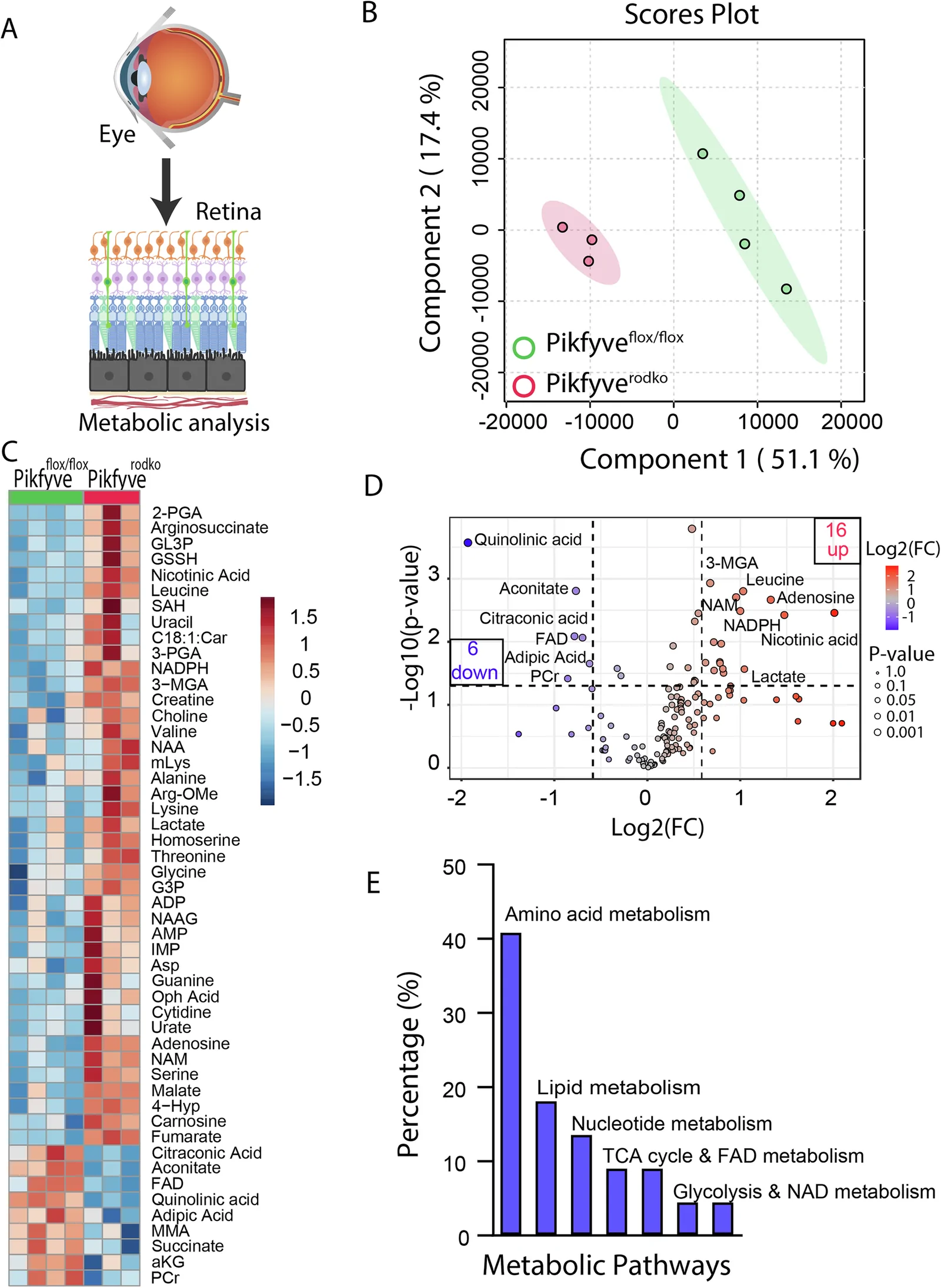
Impact of PIKfyve deficiency in rods on retinal metabolism. Retinas were harvested from 8-week-old *Pikfyve*^*flox/flox*^ and *Pikfyve*^*rodko*^ mice to examine the steady-state levels of retinal metabolites (**A**). Principal component analysis (PCA) of the samples showed a clear separation between the *Pikfyve*^*flox/flox*^ and *Pikfyve*^*rodko*^ mouse retinas (**B**). A heatmap of changed metabolites in *Pikfyve*^*rodko*^ compared to *Pikfyve*^*flox/flox*^ mouse retina (**C**). Volcano plot showing differential metabolites in *Pikfyve*^*rodko*^ compared to *Pikfyve*^*flox/flox*^ mouse retina (**D**). Horizontal line indicates changes with (*p* < 0.05). Data are mean ± *SEM, (n* = 4 *WT; n* = 3 *KO)*. The histogram shows the pathway alterations in *Pikfyve*^*rodko*^ compared to *Pikfyve*^*flox/flox*^ mouse retina (**E**).

Muscle-specific *Pikfyve* gene disruption has been shown to alter energy metabolism [36]. To evaluate how PIKfyve deficiency in rods affects retinal metabolism, we analyzed steady-state metabolite levels in 8-week-old *Pikfyve*^*flox/flox*^ and *Pikfyve*^*rodko*^ mouse retinas using LC-MS (Fig. 9A). Principal component analysis (PCA) of the samples showed a clear separation between the *Pikfyve*^*flox/flox*^ and *Pikfyve*^*rodko*^ mouse retinas (Fig. 9B). Our study showed that several metabolites were altered in *Pikfyve*^*rodko*^ mouse retinas compared to *Pikfyve*^*flox/flox*^ mouse retinas (Fig. 9C), with 16 metabolites upregulated and 6 metabolites downregulated (Fig. 9D). Based on the metabolomics data, the affected pathways in the *Pikfyve*^*rodko*^ retina include amino acid metabolism, lipid metabolism, nucleotide metabolism, the TCA cycle, FAD metabolism, glycolysis, and NAD metabolism (Fig. 9E).

Glucose starvation has been shown to induce autophagy via ULK1-mediated activation of PIKfyve [37]. Therefore, we examined the levels of key proteins involved in glucose metabolism in the RPE and retina of *Pikfyve*^*flox/flox*^ and *Pikfyve*^*rpeko*^ mice. We found significantly decreased levels of pPKM2 in 2-month-old *Pikfyve*^*rpeko*^ mouse RPE compared to *Pkm2* ^*flox/flox*^ mouse RPE (Fig. S5A). PKM2 and LDHA levels are also reduced in *the Pikfyve*^*rpeko*^ mouse RPE compared with the *Pikfyve*^*flox/flox*^ mouse; however, the difference is not statistically significant (Fig. S5A, D). We found significantly increased levels of ALDOC, Glut1, rod arrestin, and M-opsin in the retina of *Pikfyve*^*rpek*o^ mice compared to *Pikfyve*^*flox/flo*x^ mice (Fig. S5B, C, E). Rhodopsin levels are higher in the retina of *Pikfyve*^*rpeko*^ mice than in *Pikfyve*^*flox/flox*^ controls, though the difference is not statistically significant (Fig. S5B, E). This increase may result from reduced phagocytic activity of PIKfyve-deficient RPE.

## Discussion

This study first demonstrates that loss of PIKfyve in rods leads to lysosomal dysfunction and retinal degeneration, whereas loss of PIKfyve in the RPE results in lipid deposit accumulation. In *Drosophila*, defective rhodopsin trafficking results in the accumulation of late endosomes and light-dependent degeneration, underscoring the importance of efficient endolysosomal degradation for photoreceptor survival [19, 20]. These findings, along with those from our current study, suggest that the endolysosomal degradative pathway is highly conserved across invertebrates and vertebrates. Furthermore, the results highlight the critical role of PIKfyve in rod photoreceptors and the RPE functions.

In *Pikfyve*^*rodko*^ mice, rhodopsin mislocalization was evident, and the retinal phenotype resembled a lysosomal storage disorder (LSD), characterized by lysosomal vacuole accumulation and elevated lysosomal protein levels [38, 39]. LAMP-1 and LAMP-2, known biomarkers of LSDs, were also increased [38, 39]. These findings suggest that loss of PIKfyve in rod photoreceptors disrupts PI(3,5)P₂-dependent lysosomal homeostasis, leading to enlarged, dysfunctional lysosomes and subsequent degeneration. Proteins in the inner segment, misdirected rhodopsin, excess rhodopsin, or misfolded proteins are likely cleared through the endolysosomal pathway. Supporting this, loss of either one or both PIKfyve alleles in *P23H*^*het*^ mice accelerated photoreceptor degeneration, underscoring PIKfyve’s role in protein clearance and highlighting its potential as a therapeutic target. PIKfyve-generated PI(3,5)P₂ is required for the regulation of autophagy [40]. Consistent with this, previous studies show that induction of autophagy enhances the clearance of P23H rhodopsin aggregates and protects against retinal degeneration [41]. Our studies demonstrate that lysosomal dysfunction in rods precedes retinal degeneration, ultimately leading to the loss of rod structure and function in mice lacking PIKfyve in rods. Interestingly, rod-specific deletion of PIKfyve reduces RPE65 protein levels in the RPE, which may contribute to the observed decline in photoreceptor function in these mice.

Our ultrastructural studies reveal the accumulation of vacuoles in the rod inner segments, and characterization of these vacuoles in vitro shows increased Rab7 and LC3A/B levels, indicating an accumulation of amphisomes—hybrid organelles formed by the fusion of autophagosomes with late endosomes [42]. Amphisomes subsequently fuse with lysosomes to form autolysosomes or amphisomal lysosomes, facilitating degradation. Supporting this, *pikfyve* zebrafish mutants exhibit aberrant vacuolation, characterized by the accumulation of Rab7+LC3+ amphisomes in lens cells [8]. Similar lysosomal vacuolization is observed in rods of P23H knock-in mice and in *Xenopus laevis* models expressing mutant rhodopsin [43]. The P23H mutation induces chronic ER stress and persistent activation of the Unfolded Protein Response, overwhelming clearance pathways and leading to rhodopsin aggregation, trafficking defects, oxidative stress, and photoreceptor death [44, 45]. Recent studies also link PIKfyve to ER-lysosome communication, showing that its inhibition disrupts ER morphology and motility through abnormal lysosome clustering and protrudin tethering [46]. These findings highlight PIKfyve’s essential role in proteostasis and organelle homeostasis, suggesting it as a potential target to preserve photoreceptor survival.

RPE plays a vital role in preserving retinal health and vision. The endo-lysosomal pathway plays a crucial role in supporting the function and survival of RPE cells [47, 48]. This pathway is responsible for processing endosomes, phagosomes, and autophagosomes, all of which are essential for cellular clearance and homeostasis [49]. Defective lysosomal function, as observed in LSDs, has been linked to progressive retinal degeneration [47]. In the RPE, loss of PIKfyve results in increased staining of rhodopsin, LAMP1, and LC3, indicating impaired phagocytosis, lysosomal function, and autophagy. Elevated LAMP1 levels may indicate a defect in lysosomal fusion with endosomes, phagosomes, or autophagosomes.

Earlier studies have shown that Oil Red O binds to lipids accumulated in Bruch’s membrane (BrM) in patients with AMD [35]. Aging is the primary risk factor for AMD, and soft drusen, along with basal laminar deposits, are lipid-rich extracellular lesions specific to AMD. Oil Red O binding to neutral lipids indicates a significant age-related deposit in the BrM and is the first identified drusen component [35]. The RPE lacking PIKfyve shows increased Oil Red O staining, indicating the importance of PIKfyve in lipid clearance and its role in AMD prevention. Interestingly, PIKFYVE has previously been identified as one of the genes associated with AMD [50]; however, this association has not been explored further. Additional studies are needed to investigate age-related changes in PIKfyve expression in the RPE.

PIKfyve-generated PI(3,5)P₂ regulates lysosomal signaling by directly binding Raptor to control mTORC1 localization and activity [51] and by directly activating the lysosomal Ca²⁺ channel TRPML1 [52]. Through these mechanisms, PIKfyve links nutrient sensing and anabolic metabolism to TFEB-dependent regulation of autophagy [53, 54]. TRPML1-mediated lysosomal Ca²⁺ release further modulates mTORC1 signaling and promotes TFEB activation, integrating lysosomal function with metabolic control [52]. Mutations in MCOLN1, which encodes TRPML1, cause mucolipidosis type IV, a disorder characterized by neurological and ophthalmologic defects [55]. Consistently, Mcoln1⁻/⁻ mice exhibit photoreceptor thinning, lysosomal enlargement, accumulation of storage material, optic nerve degeneration, and impaired visual function [56]. Consistent with disruption of this lysosomal pathway, rod-specific loss of PIKfyve resulted in broad metabolic alterations in the retina, affecting amino acid, lipid, nucleotide, glycolytic, and TCA cycle pathways. Although mTOR signaling was not directly examined, the established roles of PI(3,5)P₂ in regulating Raptor–mTORC1 and activating TRPML1 provide a link between PIKfyve deficiency, lysosomal dysfunction, and metabolic reprogramming. Further studies are required to determine whether these changes occur through mTOR-dependent or independent mechanisms.

Apilimod is being studied for a wide range of diseases, including autoimmune disorders such as [57], rheumatoid arthritis [58], and psoriasis [59]; neurodegenerative conditions such as ALS [60]; various cancers [61, 62]; and viral infections such as COVID-19 [63] and Ebola [64]. The drug primarily inhibits PIKfyve activity, thereby altering endolysosomal trafficking [65, 66] and cytokine release [59]. This inhibition can decrease inflammatory cytokine release and disrupt intracellular trafficking—key mechanisms that contribute to its antiviral and anticancer effects. However, this exact mechanism raises significant safety concerns, as PIKfyve inhibition disrupts lysosomal function, impairs autophagy, and can compromise immune function. Our study highlights that retinal photoreceptors and RPE cells are particularly vulnerable to PIKfyve loss, as these cells depend on intact endolysosomal pathways for the turnover of photoreceptor outer segments and protein quality control. Thus, our work provides crucial mechanistic insights into potential retinal risks associated with systemic PIKfyve inhibition and establishes a foundation for evaluating therapeutic opportunities and safety considerations for Apilimod and related compounds. Consistent with our findings, a recent study showed that both gene editing and pharmacological inhibition of PIKfyve with apilimod in zebrafish lead to vacuole formation in photoreceptors and the RPE [67], underscoring the retinal consequences of PIKfyve disruption.

In summary, loss of PIKfyve disrupts PI(3,5)P₂-dependent lysosomal homeostasis, impairing the clearance of misfolded and mislocalized proteins, and disturbing organelle communication. These defects lead to photoreceptor degeneration and resemble LSDs. Evidence from the P23H model further underscores PIKfyve’s role in maintaining proteostasis, as reduced activity accelerates degeneration. Together, our results demonstrate that PIKfyve-generated PI(3,5)P₂ is crucial for regulating the endolysosomal pathway in photoreceptors and RPE cells, suggesting that enhancing PIKfyve function may provide neuroprotection in retinal diseases.

Importantly, a parallel study in zebrafish showed that both genetic disruption and pharmacological inhibition of PIKfyve with apilimod produced RPE and rod phenotypes [67] that complement and are consistent with our findings. Together, these results indicate that the retinal defects arise largely from cell-autonomous loss of PIKfyve and support a conserved role for PIKfyve in maintaining retinal endolysosomal homeostasis.

### Limitations of this study

Our evaluation of Apilimod was limited to in vitro experiments, and its effects on the retina in vivo remain to be determined. We have not yet completed a time-course evaluation of RPE in mice lacking PIKfyve in rods; these studies are currently underway.

## Materials and methods

### Animals

All animal procedures complied with the ethical standards outlined in the ARVO Statement for the Use of Animals in Ophthalmic and Vision Research and adhered to the NIH Guide for the Care and Use of Laboratory Animals. Experimental protocols were approved by the Institutional Animal Care and Use Committee (IACUC) at the University of Oklahoma Health Sciences Center. Breeding colonies of BEST1-Cre (Jax #017557), RiboTag (Jax #029977), P23H (Jax #017628), and floxed Pikfyve (Jax #029331) mice were obtained from The Jackson Laboratory (Bar Harbor, ME). Rhodopsin-Cre mice were kindly provided by Dr. Ching-Kang Jason Chen at Baylor College of Medicine (Houston, TX). All mice were housed in our institutional animal facility under a controlled 12-h light/dark cycle (40–60 lux). Animals were screened and confirmed negative for rd1 and rd8 retinal degeneration mutations. To reduce experimental bias, mice were randomly assigned to groups matched for sex, age, and genetic background. Litters were pooled to prevent litter-specific confounding effects.

### Generation of Rod- and RPE-specific Pikfyve knockout mice

Rod- and RPE-specific conditional Pikfyve knockout mice were generated using the Cre-loxP system. Floxed Pikfyve mice were crossed with transgenic mice expressing Cre recombinase under the rod-specific rhodopsin promoter (rod-Cre Pikfyve-KO) or the RPE-specific bestrophin promoter (RPE-Cre Pikfyve-KO). In both models, Cre-mediated excision of exon 6 resulted in tissue-specific deletion of Pikfyve. Genotyping was performed by PCR using tail DNA. Rod-Cre (opsin-Cre) yielded a 500 bp product, and RPE-Cre yielded a 158 bp product. Floxed Pikfyve alleles produced a 421 bp band for the wild-type allele, both 421 bp and 500 bp bands for heterozygotes, and a 500 bp band for the mutant allele. Primer sequences are listed in Supplementary Table 1.

### Transmission electron microscopy

TEM was carried out as described earlier [68]. Mouse eyes were collected after brief CO₂ exposure followed by cervical dislocation. Eyes were enucleated, rinsed, and immersion-fixed in 100 mM cacodylate buffer (pH 7.5) containing 2.5% glutaraldehyde and 2% paraformaldehyde. The cornea was punctured to allow fixative penetration, and the anterior segment (cornea, iris, and lens) was removed to prepare eyecups. Eyecups were fixed at room temperature and stored at 4 °C until further processing. Eyecups were dissected into small pieces, washed in 0.1 M cacodylate buffer, post-fixed in 2% osmium tetroxide for 1 h on ice, and incubated overnight in 1% uranyl acetate at 4 °C. Samples were dehydrated through graded ethanol, treated with propylene oxide, and infiltrated with Araldite/Embed resin before embedding and polymerization at 60 °C. Semithin sections (0.5–2 µm) were cut, stained with Richardson’s stain, and examined by light microscopy. Ultrathin sections (50–100 nm) were cut with a diamond knife, mounted on formvar-coated grids, air-dried, and examined by TEM.

### Statistical analysis

Sample sizes were calculated using power analyses. To eliminate bias, animals were randomly assigned to groups by sex, age, and strain, and litters were mixed to avoid litter bias. Animals were genotyped, assigned unique identifiers, and randomly selected to ensure that study personnel remained blinded during experimentation and analysis. All statistical analyses were performed with GraphPad Prism version 7.3. No data points were excluded from any analysis. The selection of statistical tests was based on experimental design. Before analysis, the normality of each dataset was checked using multiple tests, including the Anderson-Darling, D’Agostino-Pearson, Shapiro-Wilk, and Kolmogorov-Smirnov tests, to determine if the data followed a normal distribution. For datasets that failed normality tests, non-parametric methods—specifically, multiple Mann-Whitney *U* tests—were used to compare the groups. To adjust for multiple comparisons and control the false discovery rate, the two-stage Benjamini-Hochberg-Yekutieli procedure was applied with a *Q*-value threshold of 1%. For normally distributed data, comparisons between two groups used parametric tests with Welch’s correction to account for unequal variances. Statistical significance was based on *p*-values. One-way ANOVA was used to compare three or more independent groups, while two-way ANOVA evaluated the interaction between two categorical variables on a continuous outcome.

### Other methods

TRAP and the generation of rod-, cone-, RPE-, Müller glia-, and RGC-specific Rpl22 mice have been previously described [15]. Primer sequences used for *Pikfyve* expression analysis and normalization to *Rpl37* and *Rpl38* are listed in Supplementary Table 2. ERG, tissue histology, Western blotting, and immunohistochemical staining were performed according to previously established protocols [69]. The antibodies used in this study are listed in Supplemental Table 3. Targeted metabolomics was performed using LC-MS as described in [70]. We added Nicotinamide-D4 as the internal standard for quality control.

## Supplementary information


Uncropped Western blots
Supplmentary Materials


## Data Availability

All data generated or analyzed during this study are included in this manuscript. The metabolomics LC/MS Mass Spectrometry data has been deposited in the MassIVE Repository under Reference number MSV000100803.
