# Supplementary material for: PIKfyve preserves endolysosomal function in photoreceptors and RPE cells to maintain retinal integrity
Source: Cell Death Dis. 2026 May 16;17(1):759. doi: 10.1038/s41419-026-08855-2 (PMC13522625; doi:10.1038/s41419-026-08855-2)
Supplement: Supplementary file 1 [file 41419_2026_8855_MOESM2_ESM.docx]

**Figure S1. Original TEM micrographs related to Figure 5.** Original, unprocessed TEM images (**A-H**) of photoreceptor inner and outer segment regions from *Pikfyve^flox/flox^* and *Pikfyve^rodko^* retinas. The images were only resized to fit the figure layout and were not cropped or otherwise modified. Scale bars: A=6 µm; B=6 µm; C=4 µm; D=6 µm; E=4 µm; F=4 µm; G=1 µm; H=1 µm.

**Figure S2. Immunoblot analysis of RPE proteins in *Pikfyve^rodko^* mice.** RPE protein lysates from *Pikfyve^flox/flox^* and *Pikfyve^rodko^* mice were analyzed by immunoblotting using antibodies against RPE65, LAMP1, LAMP2, LC3A/B, and actin (A). Band intensities were quantified and normalized to actin (B). Data are presented as mean ± *SEM (n = 3).*

**Figure S3. TEM images of the RPE–outer segment interface in *Pikfyve^flox/flox^* (A) and *Pikfyve^rodko^* (B) retinas.** The images were resized solely for figure layout and were not cropped or otherwise altered. Scale bars: A = 10 µm; B = 6 µm. **N**, nucleus; **M**, melanosomes; **L**, lipofuscin granules.

**Figure S4. Functional characterization of *Pikfyve^rpeko^* mice.** ERG was performed on 24-week-old *Pikfyve^flox/flox^* and *Pikfyve^rpeko^* mice to examine the amplitudes of the scotopic a-wave (A), scotopic b-wave (B), and photopic b-wave (C). Data are mean ± *SEM (n=8).*

**Figure S5. Immunoblot analysis of metabolic, photoreceptor, and glial proteins in RPE and retina of *Pikfyve^rpeko^* mice.** Protein lysates from RPE (A) and retina (B, C) from *Pikfyve^flox/flox^* and *Pikfyve^rpeko^* were immunoblotted with ALDOC, PKM2, pPKM2, PKM1, LDHA, LDHB, Rpe65, Glut1, Rhodopsin, rod arrestin, GFAP, GS, M-opsin, cone arrestin, and actin antibodies. Protein densities from RPE (D) and retina (E) were normalized to actin. Data are mean ± *SEM, (n = 4).*

**Table S1. Primer sequences used for genotyping rod- and RPE-specific Pikfyve conditional KO mice**

| **Gene** | **Forward Primer** | **Reverse Primer** |
| --- | --- | --- |
| Rod-Cre | TCAGTGCCTGGAGTTGCGCTGTGG | CTTAAAGGCCAGGGCCTGCTTGGC |
| BEST1-Cre | GGC ACT GGC CAC AGA GTC | GGT GTA CGG TCA GTA AAT TGG A |
| Pikfyve floxed | GCC TGA GTT CTG AGA GTG AGT G | CAC TGG CTA TCT GGC ATC C |

**Table S2: Real-time PCR primers to quantify the expression of mouse *Pikfyve in Rod, Cone, RPE,* Müller glia, and RGC**

| **Gene** | **Forward Primer** | **Reverse Primer** |
| --- | --- | --- |
| *Pikfyve* | GATTCATCCGGATTCCTCAA | TAGCCTGGGGACTGACAGAT |
| *Rpl37* | CGGGACTGGTCGGATGAG | TCACGGAATCCATGTCTGAATC |
| *Rpl38* | CGCCATGCCTCGGAAA | CCGCCGGGCTGTCAG |

| **Table S3. Antibodies used for immunoblotting and immunofluorescence (IF) Analysis** | | | | |
| --- | --- | --- | --- | --- |
| **Antibody** | **Host species** | **Dilution** | **Manufacturer** | **Catalog number** |
| PIKFyve | Mouse | 1:1000 | Developmental Studies Hybridoma Bank (DSHB) | 1H2-s |
| PIKFyve | Mouse | 1:1000 | Developmental Studies Hybridoma Bank (DSHB) | 2B2-s |
| LDHA | Rabbit | 1:1000  1:50 (IF) | Proteintech | 21799-1-AP |
| LDHB | Rabbit | 1:1000  1:50 (IF) | Proteintech | 14824-1-AP |
| Pde6β | Mouse | 1:1000  1:25 (IF) | Santa Cruz | SC-377486 |
| Rhodopsin | Mouse | 1:1000  1:50 (IF) | In-house | Gift from Dr. Jim McGinnis (OUHSC) |
| Rod-Arrestin | Mouse | 1:1000  1:500 (IF) | In-house | Gift from Dr. Paul Hargrave (University of Florida) |
| M-opsin | Rabbit | 1:1000  1:100 (IF) | Millipore Sigma | AB5405 |
| S-opsin | Rabbit | 1: 100 (IF) | Millipore Sigma | ABN1660-1 |
| Cone-Arrestin | Rabbit | 1:1000 | Millipore Sigma | AB15282 |
| Actin | Mouse | 1:1000 (IB) | Thermo Fisher Scientific | MA1-744 |
| Glutamine synthetase (GS) | Mouse | 1:1000  1:50 (IF) | Abcam | Ab64613 |
| Glial fibrillary acid protein (GFAP) | Rabbit | 1:100 (IF) | Dako | 20334 |
| PKM1 | Rabbit mAb | 1:1000  1:50 (IF) | Cell Signaling Technology | 7067 |
| PKM2 | Rabbit mAb | 1:1000  1:100 (IF) | Cell Signaling Technology | 4053 |
| Phospho-PKM2 (Tyr105) | Rabbit | 1:1000  1:50 (IF) | Cell Signaling Technology | 3827 |
| Aldolase C | Mouse | 1:1000  1:100 (IF) | EnCor Biotechnology, Inc. | MCA-4A9 |
| Glut1 | Rabbit | 1:1000  1:25 (IF) | Novus Biologicals | NB110-39113 |
| Rpe65 | Rabbit | 1:1000 | In-house | Gift from Dr. Jian-Xing Ma |
| Rab5A | Rabbit | 1:1000 | Cell Signaling Technology | 46449 |
| Rab7 | Rabbit | 1:1000 | Cell Signaling Technology | 9367 |
| LC3A/B | Rabbit | 1:1000 | Cell Signaling Technology | 12741 |
| LAMP1 | Rat | 1:1000 1:100 (IF) | Developmental Studies Hybridoma Bank (DSHB) | 1D4B |
| LAMP2 | Rat | 1:1000 1:100 (IF) | Developmental Studies Hybridoma Bank (DSHB) | GLA2A7 |
